# Supplementary material for: Prevalence and molecular characterization of antibiotic resistance and associated genes in Klebsiella pneumoniae isolates: A clinical observational study in different hospitals in Chattogram, Bangladesh
Source: PLoS One. 2021 Sep 10;16(9):e0257419. doi: 10.1371/journal.pone.0257419 (PMC8432802; doi:10.1371/journal.pone.0257419)
Supplement: S1 Raw images — (PDF) [file pone.0257419.s003.pdf]

Gel images of …

NDM-1, SHV-11, uge (Figure 3)

Plasmid size (Figure 6)

NDM-1

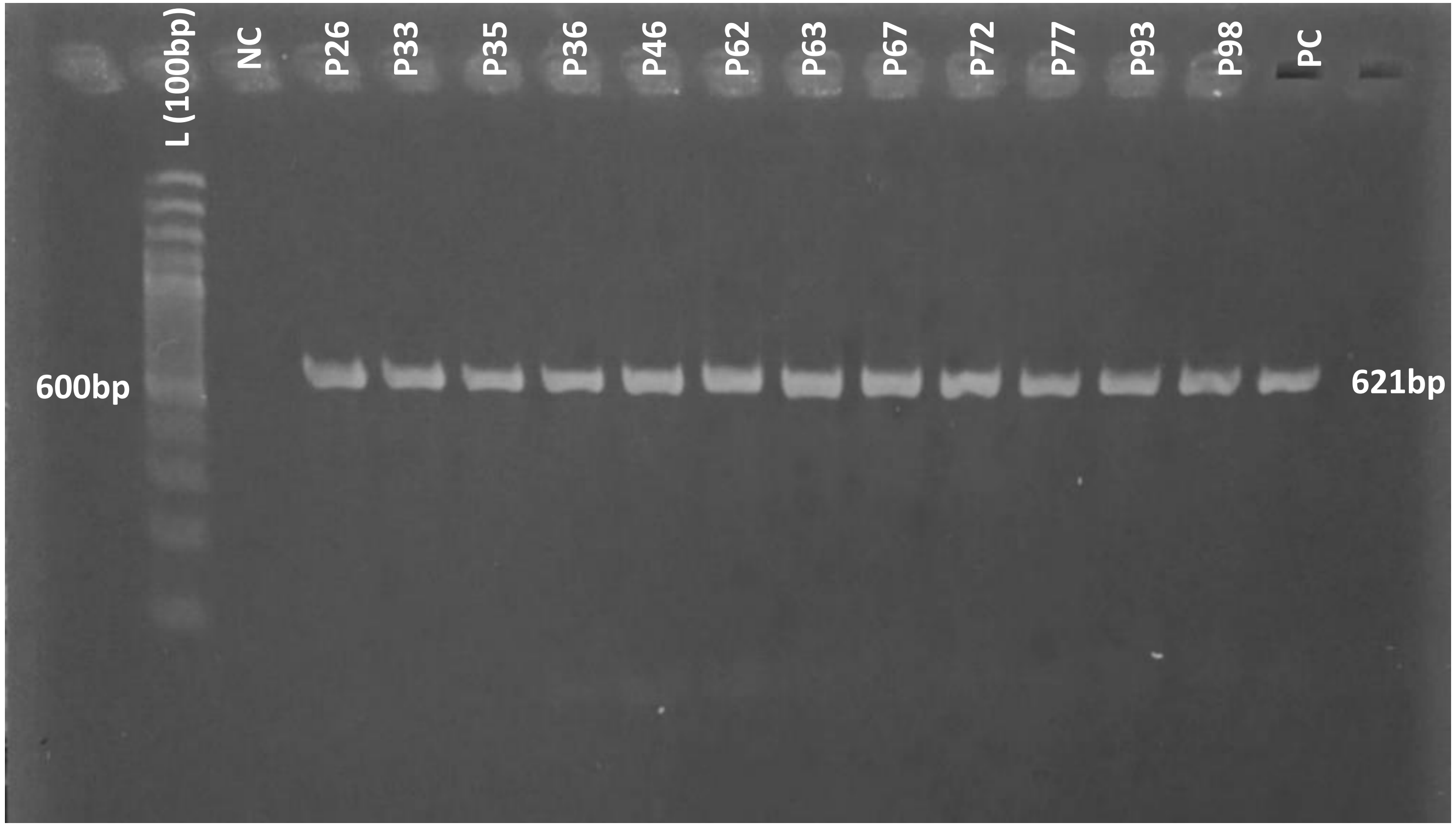
L (100bp)
NC
P26
P33
P35
P36
P46
P62
P63
P67
P72
P77
P93
P98
PC
600bp
621bp

SHV-11

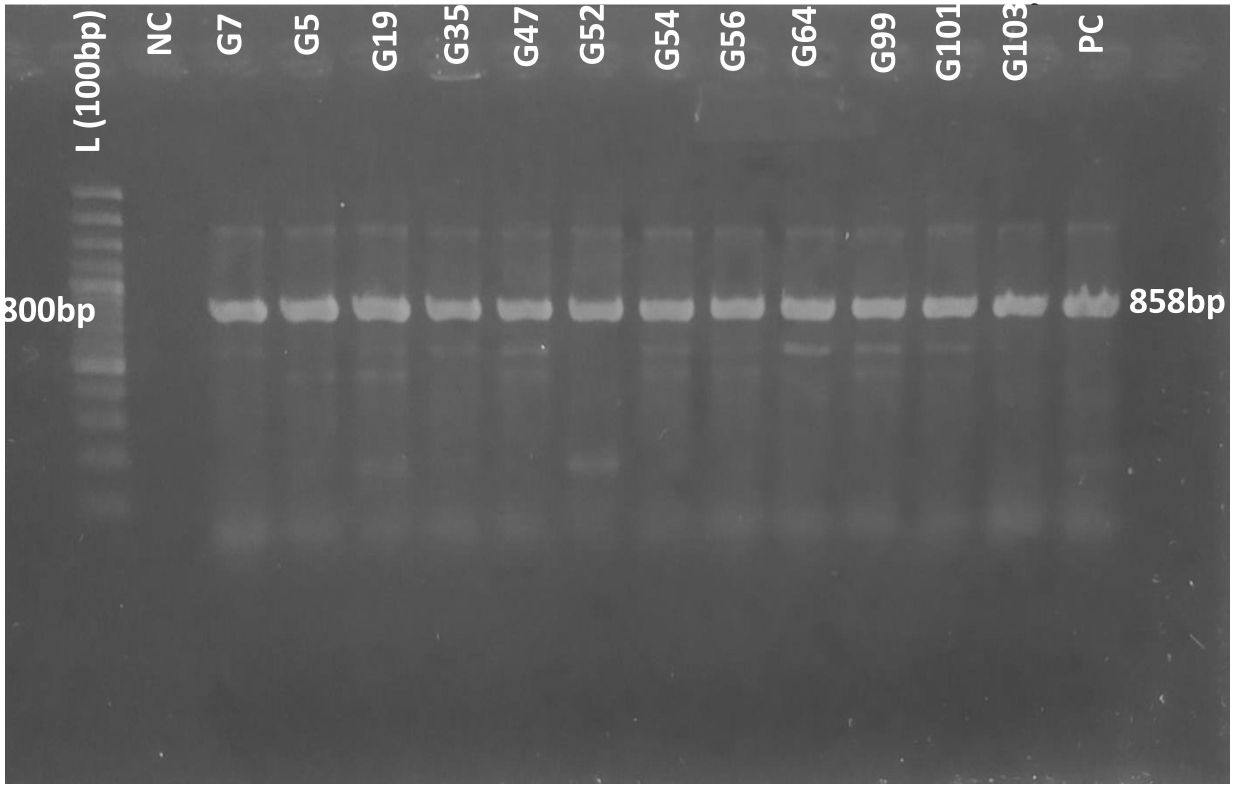

L (100bp)
NC
G7
G5
G19
G35
G47
G52
G54
G56
G64
G99
G101
G103
PC
800bp
858bp

UGE

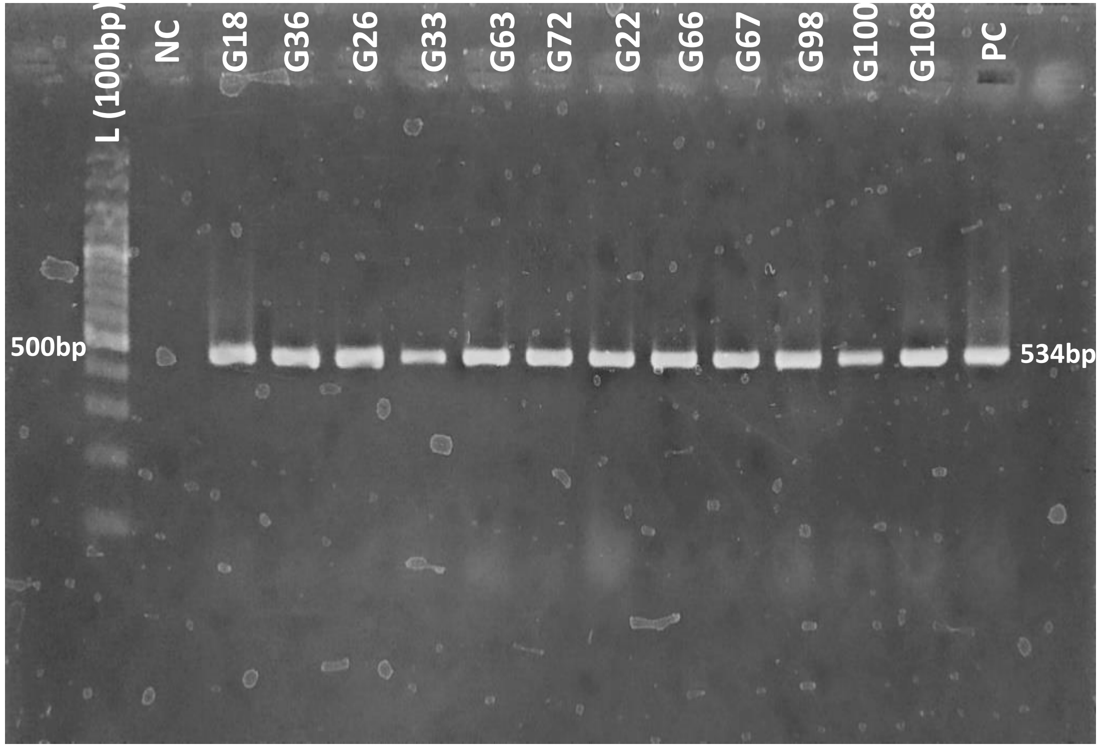

L (100bp)
NC
G18
G36
G26
G33
G63
G72
G22
G66
G67
G98
G100
G108
PC
500bp
534bp

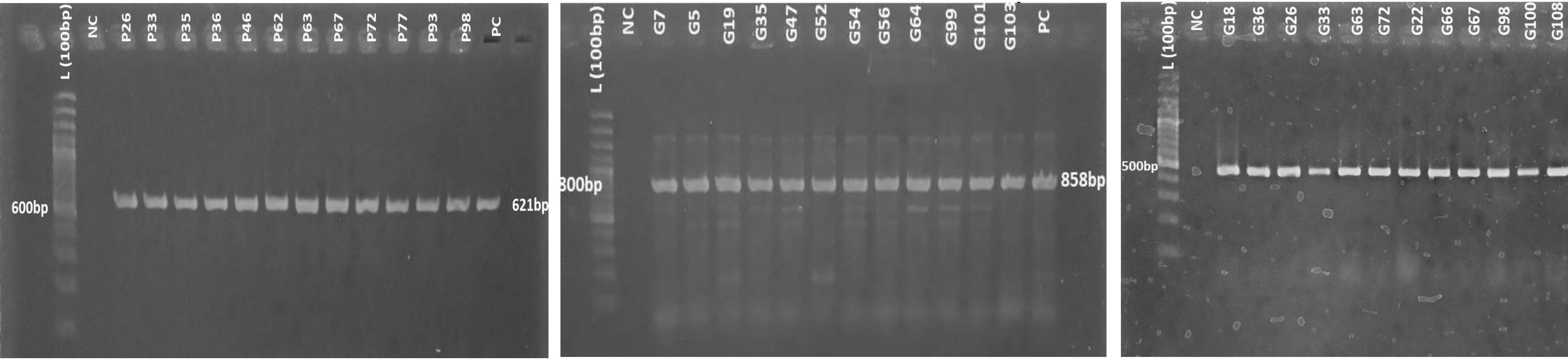

A. *bla*$_{NDM-1}$ **B. *bla*$_{SHV-11}$** **C. uge**

Figure 3: Amplification and confirmation of blaNDM-1, blaSHV-11, and uge genes by polymerase chain reaction. PCR products were visualized in 1.5% agarose gel using ethidium bromide staining (0.5%). L indicates Ladder (100 bp); NC as a negative control(Nuclease free water); PC indicates positive control, K. pneumoniae ATCC70603. (A) samples representing bands with 621bp were blaNDM-1 positive amplicons similarly, (B) samples were blaSHV-11 positive amplicons with 858bp, (C) samples were uge positive amplicons having 534bp.

Plasmid profiling (Figure 6)

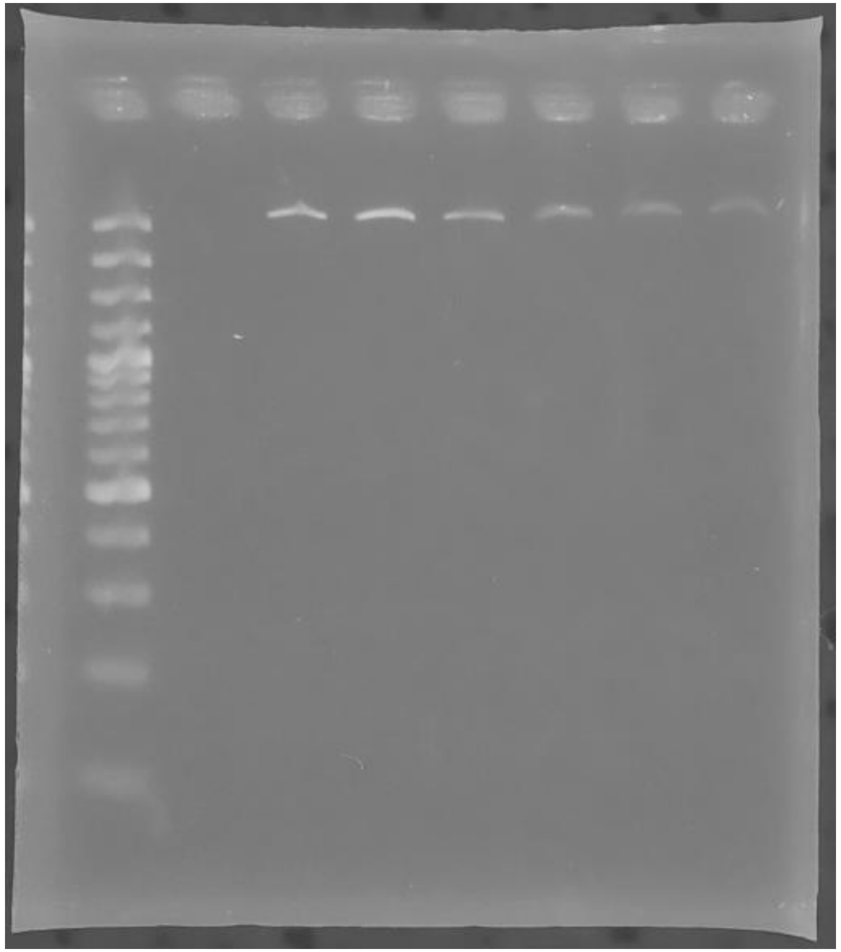

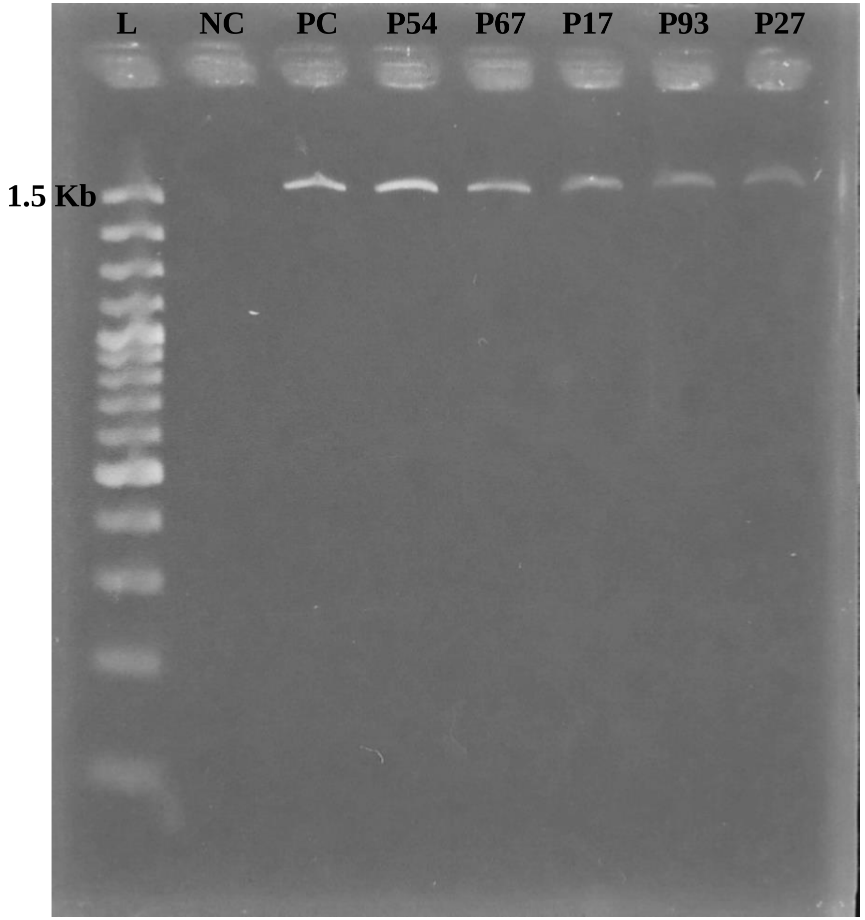

Figure 6: Detection of plasmid by Agarose gel electrophoresis.
