# Supplementary material for: Prevalence and molecular characterization of antibiotic resistance and associated genes in Klebsiella pneumoniae isolates: A clinical observational study in different hospitals in Chattogram, Bangladesh
Source: PLoS One. 2021 Sep 10;16(9):e0257419. doi: 10.1371/journal.pone.0257419 (PMC8432802; doi:10.1371/journal.pone.0257419)
Supplement: Attachment — Submitted filename: Reviewer recommendations.pdf [file pone.0257419.s004.pdf]

Prevalence of antibiotic resistance and associated genes among clinical isolates of Klebsiella pneumoniae in the southern region of Bangladesh

Reviewer's recommendations

Line 84. When referring to the classes of antibiotics, ESBL is placed, which is a type of resistance and not a class of antibiotic. It is suggested to place beta-lactams as a class of antibiotics

Line 107. It is not necessary to capitalize Extended

Line 154. It is not necessary to capitalize Standard

Lines 155 and 156. It is suggested to place the size of the Petri dishes used since 8 discs should not be placed in boxes of 100 x 10 (15) but a maximum of 6 according to the CLSI recommendations.

Line 156. Why do you use Ampicillin disc for Klebsiella? Has natural resistance to this antibiotic

Line 157. Why do you place an azithromycin disk? This is used in S.Typhi or Shigella

The list of antibiotics used for the test does not include any beta-lactamase inhibitor. Why?

A resistance analysis should be done according to the origin of the patient. Inpatients or outpatients

Line 214. What was the selection methodology for resistant Klebsiella?

Line 245, 246. The names of the bacteria must be written in italics

Line 252. Correct typing error

Line 263. Place a period after the word role

Line 268. Correct typing error

Line 280. When speaking of a reservoir, it refers to a normal growth site. It is suggested to modify the word by site of infection

Line 282. The year of the reference should be in parentheses

Line 284 to 286. How did they show that they are treating the same Klebsiella clone as the patients infections?

Line 288. Write in italics Enterobacteriacea

Line 304. Remove the quotation mark after patients

Line 309. Put the year in parentheses

Line 315. Place the year in parentheses in the reference

Line 323. Enter the year of the reference

Line 327. Put the year in parentheses

Line 345. Correct typing error

Line 347. The study was carried out in two healthcare centers in Chattogram. The results could not be generalized to the southern Bangladesh region

Line 355. Correct typing error

Figure 1A. Azithromycin should not be listed. Amox + Clav is not described in materials

Figures 1-4 are repeated in the document
