# Supplementary material for: Prevalence and molecular characterization of antibiotic resistance and associated genes in Klebsiella pneumoniae isolates: A clinical observational study in different hospitals in Chattogram, Bangladesh
Source: PLoS One. 2021 Sep 10;16(9):e0257419. doi: 10.1371/journal.pone.0257419 (PMC8432802; doi:10.1371/journal.pone.0257419)
Supplement: Attachment — Submitted filename: Reviewer recommendations 2.pdf [file pone.0257419.s006.pdf]

Prevalence of antibiotic resistance and associated genes among clinical isolates of Klebsiella pneumoniae in the southern region of Bangladesh

Reviewer's recommendations

Line 41. Klebsiella in italics

Line 82. Place the year in parentheses

Line 87. Enterobacteriaceae in italics

Lines 147 to 150. They are proper names of the units and the first letter of each word should be capitalized (unify, some are capitalized while others are not)

Line 170. Klebsiella pneumoniae in italics

Line 186 to 191. When describing the primers used, it is not necessary to place F or R before the sequence, taking into account that they already indicate whether it is Forward or Reverse. It is recommended to place the name of the amplified gene in the case of the third pair of primers. If the 5'-3 'orientation is specified at the beginning of all primers, the extremities of each sequence could be eliminated. All this in order to improve the presentation and facilitate reading.

Line 192 the reference has an asterisk. Can't find where this asterisk points to

Line 265 Correct writing ceftriaxoMe

Line 277 separate Figure 3; Table2. To avoid confusion

Figure 4. It would be interesting for the authors to mention the clonality of these analyzes. Try to hypothesize against this. The analysis in sewerage water and bed trails from samples obtained from the neonatal Ward is mentioned. Did you make comparative analyzes between the KPN of samples with those of fomites? Was cross contamination found ?. It is recommended to discuss and conclude regarding the clonality found, this data is of interest

Line 365 to 370. Fomite analysis is not mentioned in materials and methods.

Line 404. Place the year in parentheses

Line 429. Genus and bacterial species in italics
