# Supplementary material for: Prevalence and molecular characterization of antibiotic resistance and associated genes in Klebsiella pneumoniae isolates: A clinical observational study in different hospitals in Chattogram, Bangladesh
Source: PLoS One. 2021 Sep 10;16(9):e0257419. doi: 10.1371/journal.pone.0257419 (PMC8432802; doi:10.1371/journal.pone.0257419)
Supplement: Attachment — Submitted filename: Reviewer recommendations 3.pdf [file pone.0257419.s008.pdf]

Prevalence of antibiotic resistance and associated genes among clinical isolates of Klebsiella pneumoniae in the southern region of Bangladesh

Reviewer's recommendations

After reviewing the requested changes, the article is considered ready for publication. Congratulations to the authors for their effort and dedication.
