# Supplementary material for: Prevalence and molecular characterization of antibiotic resistance and associated genes in Klebsiella pneumoniae isolates: A clinical observational study in different hospitals in Chattogram, Bangladesh
Source: PLoS One. 2021 Sep 10;16(9):e0257419. doi: 10.1371/journal.pone.0257419 (PMC8432802; doi:10.1371/journal.pone.0257419)
Supplement: Attachment — Submitted filename: Response to the reviewer.docx [file pone.0257419.s007.docx]

**Prevalence and molecular characterization of antibiotic resistance and associated genes in Klebsiella pneumoniae isolates: A clinical observational study in different hospitals in Chattogram, Bangladesh**

**Reviewer’s recommendations**

Line 41. Klebsiella in italics

Response: We would like to thank the reviewer for this suggestion. We have rechecked the manuscript and corrected the error.

Line 82. Place the year in parentheses

Response: We would like to thank the reviewer for this suggestion. We have rechecked the manuscript and corrected the error.

Line 87. Enterobacteriaceae in italics

Response: We would like to thank the reviewer for this suggestion. We have rechecked the manuscript and corrected the error.

Lines 147 to 150. They are proper names of the units and the first letter of each word should

be capitalized (unify, some are capitalized while others are not)

Response: We would like to thank the reviewer for this suggestion. We have rechecked the manuscript and edited accordingly.

Line 170. Klebsiella pneumoniae in italics

Response: We would like to thank the reviewer for this suggestion. We have rechecked the manuscript and corrected the error.

Line 186 to 191. When describing the primers used, it is not necessary to place F or R before the sequence, taking into account that they already indicate whether it is Forward or Reverse. It is recommended to place the name of the amplified gene in the case of the third pair of primers. If the 5'-3' orientation is specified at the beginning of all primers, the extremities of each sequence could be eliminated. All this in order to improve the presentation and facilitate reading.

Response: We would like to thank the reviewer for this suggestion. Changes have been made accordingly.

Line 192 the reference has an asterisk. Can't find where this asterisk points to

Response: We would like to thank the reviewer for this suggestion. We have rechecked the manuscript and corrected the error.

Line 265 Correct writing ceftriaxoMe

Response: We would like to thank the reviewer for this suggestion. We have rechecked the manuscript and corrected the error.

Line 277 separate Figure 3; Table2. To avoid confusion

Response: We would like to thank the reviewer for this suggestion. We have rechecked the manuscript and edited accordingly.

Figure 4. It would be interesting for the authors to mention the clonality of these analyzes. Try to hypothesize against this. The analysis in sewerage water and bed trails from samples obtained from the neonatal Ward is mentioned. Did you make comparative analyzes between the KPN of samples with those of fomites? Was cross contamination found ?. It is recommended to discuss and conclude regarding the clonality found, this data is of interest

Response: We agree with the reviewer that clonality should be monitored. However, the fomites were assessed only to check the presence of KPN. It can give an idea if there is a chance of transmission / cross contamination from fomite to the patients. But this needs more samples from fomites. And an in depth molecular study on fomite and source of contamination is needed. We are working on the molecular identification of fomite and reservoir of the pathogen in the next phase of this study and that is a separate project. Considering the need of in depth analysis, we have excluded this part from the result and discussion of the modified manuscript. We believe this will help the readers to keep focus on the prevalence of antibiotic resistance in *Klebsiella* isolates from the clinic samples.

Line 365 to 370. Fomite analysis is not mentioned in materials and methods.

Response: Information regarding fomite has been excluded from the modified manuscript and the reason for this is explained in the above response.

Line 404. Place the year in parentheses

Response: We would like to thank the reviewer for this suggestion. We have rechecked the manuscript and corrected the error.

Line 429. Genus and bacterial species in italic

Response: We would like to thank the reviewer for this suggestion. We have rechecked the manuscript and corrected the error.
